# Low-energy extracorporeal shock wave therapy for a model of liver cirrhosis ameliorates liver fibrosis and liver function

**DOI:** 10.1038/s41598-020-58369-w

**Published:** 2020-02-12

**Authors:** Naoto Ujiie, Toru Nakano, Masato Yamada, Chiaki Sato, Chikashi Nakanishi, Fumiyoshi Fujishima, Kenta Ito, Tomohiko Shindo, Hiroaki Shimokawa, Takashi Kamei

**Affiliations:** 1 Division of Advanced Surgical Science and Technology, Tohoku University Graduate School of Medicine, Sendai, Miyagi Japan; 2 Division of Gastroenterologic and Hepatobiliarypancreatic Surgery, Tohoku Medical and Pharmaceutical University Hospital, Sendai, Miyagi Japan; 3 Department of Anatomic Pathology, Tohoku University Graduate School of Medicine, Sendai, Miyagi Japan; 4 Department of Cardiovascular Medicine, Tohoku University Graduate School of Medicine, Sendai, Miyagi Japan

**Keywords:** Liver cirrhosis, Experimental models of disease

## Abstract

Low-energy extracorporeal shock waves (LESW) have been studied as a new treatment for angina pectoris and several ischemic diseases because of its effect on angiogenesis and inhibition of fibrosis of the heart. The effect of LESW on fibrosis in liver cirrhosis has not been studied. The aim of this study was to verify the amelioration of liver fibrosis by LESW and elucidate its mechanisms in a rat model of drug-induced liver cirrhosis. Male Wistar rats aged 7 weeks were injected with carbon tetrachloride intraperitoneally twice a week for 12 weeks. Eight rats underwent LESW therapy (0.25 mJ/mm^2^, 4 Hz, 1000 shots) under general anesthesia (shock wave group). Seven rats only underwent general anesthesia (control group). Quantitative analysis showed that the area of fibrosis in the shock wave group was significantly reduced compared with the control group (11,899.9 vs. 23,525.3 pixels per field, *p* < 0.001). In the shock wave group, the mRNA expression of transforming growth factor (TGF)-β1 was significantly suppressed (0.40-fold, *p* = 0.018) and vascular endothelial growth factor-B was significantly increased (1.77-fold, *p* = 0.006) compared with the control group. Serum albumin was significantly higher in the shock wave group than in the control group (3.0 vs. 2.4 g/dl, *p* = 0.025). Aspartate aminotransferase/alanine aminotransferase ratio decreased by LESW compared with the control group (1.49 vs. 2.04, *p* = 0.013). These results suggest that LESW therapy ameliorates liver fibrosis by reducing the expression of TGF-β1 and increasing the expression of angiogenic factors, and improves hepatic function.

## Introduction

Liver cirrhosis is a life-threatening problem; a considerable proportion of patients with liver cirrhosis progress to liver failure and hepatocellular carcinoma^1^. Current medical therapy for liver cirrhosis is performed for the purpose of improving liver function^2^. An effective treatment for liver fibrosis has not been developed; therefore, establishment of a breakthrough therapy with sufficient efficacy and safety is desired.

Low-energy extracorporeal shock waves (LESW), with about 10% of the energy density used for urolithiasis, has been widely and safely used for experimental treatment of various diseases, such as ischemic cardiovascular disorders and peripheral artery disease^3–5^. The efficacy of LESW for each of these pathological lesions has been reported. LESW therapy increases the expression of vascular endothelial growth factor (VEGF) in cultured endothelial cells^6^. In addition, LESW therapy has anti-fibrotic effects in the left ventricle after acute myocardial infarction by suppressed transforming growth factor (TGF)-β1 expression, which is known to promote left ventricular fibrosis^7^. Therefore, it is hypothesized that LESW for liver cirrhosis might promote angiogenesis in the liver and ameliorate hepatic fibrosis.

To the best of our knowledge, there has been no report of the effects of LESW on liver cirrhosis. The present study aimed to verify and elucidate the mechanism of the effects of LESW on the liver, such as hepatic function, hepatic fibrosis and angiogenesis in a rat model of liver cirrhosis.

## Materials and Methods

### Animals

Specific pathogen-free male Wistar rats aged 7 weeks, weighing approximately 200 g, were purchased from Kumagai-shigeyasu Co. Ltd. (Sendai, Japan). All rats received humane care, were reared separately in ventilated cages, maintained in a temperature-controlled room under a 12-hour dark/light cycle, allowed free access to water and laboratory chow, and housed for several days prior to the experiments.

### Liver cirrhosis model in the rat

The rats were injected with carbon tetrachloride intraperitoneally as a 50% (vol/vol) solution in olive oil at a dose of 2 mL/kg body weight twice a week for 12 weeks to produce a liver cirrhosis model rat, as described previously^8^. Fifteen liver cirrhosis model rats were produced, and F3 and F4 in new Inuyama classification are produced at approximately the same rate described above method. Four days after the last injection of carbon tetrachloride, the rats were randomly divided into shock wave (*n* = 8) and control (*n* = 7) groups.

To investigate the normal condition, three rats were left untreated and served as untreated controls (untreated group).

### LESW therapy

The rats in the shock wave group were administered 0.15 mg/kg of medetomidine hydrochloride (Nippon Zenyaku Kogyo Co., Ltd., Koriyama, Japan), 2 mg/kg of midazolam (Teva Pharma Japan Inc., Nagoya, Japan), and 2.5 mg/kg of butorphanol tartrate (Meiji Seika Pharma Co., Ltd., Tokyo, Japan) via subcutaneous injection for general anesthesia. Then they were treated with LESW using a commercially available shock wave generator (Duolith® SD-1, Storz Medical AG, Tägerwilen, Switzerland). The shock waves were administered at 0.25 mJ/mm^2^, 4 Hz, 200 shots/spot, with five spots from the epigastrium to the right hypochondrium. The rats in the control group received the same procedures but without LESW treatment.

### Sample collection

Four days after the intervention, several samples were taken with laparotomy under general anesthesia with 0.15 mg/kg of medetomidine hydrochloride, 2 mg/kg of midazolam, and 2.5 mg/kg of butorphanol tartrate via subcutaneous injection. Blood samples were collected from the inferior vena cava. The liver was removed and cut into small pieces. Some pieces of left medial lobe of the liver were fixed in 10% neutral buffered formalin or zinc solution (IHC Zinc Fixative, Nippon Becton Dickinson Co., Ltd., Tokyo, Japan) for histological analysis. The liver other than the left medial lobe were snap-frozen for RNA extraction.

### Histological analysis and quantification of liver fibrosis

The liver was fixed in 10% neutral buffered formalin, then stained with Elastica-Masson. The extent of fibrosis was quantified by binarizing to fibrous or not fibrous area using Win ROOF software (version 6.3.0, Mitani Corporation, Fukui, Japan) in 10 microscopic fields (×100) per specimen in all rats from the shock wave, control and untreated groups. The quantified fibrous area was averaged for each group, and results are expressed as mean value of pixel ± standard error of the mean.

### Immunohistochemistry

The liver was fixed in 10% neutral buffered formalin, then immunostained with anti-TGF-β1 antibody (ab64715, Abcam, Tokyo, Japan). The number of TGF-β1-positive hepatic cells was counted in 10 randomly selected high-power microscopic fields (×400), and the expression rate of TGF-β1 was calculated by dividing the number of TGF-β1-positive hepatic cells by the number of all hepatic cells in a high-power microscopic field (×400). The quantified expression rate of TGF-β1 was averaged for each group, and results are expressed as mean value ± standard error of the mean.

The liver was fixed in zinc solution, then immunostained with anti-CD31 antibody (sc-1506, Santa Cruz Biotechnology, Dallas, Texas, U.S.A.). The number of CD31-positive vessels/field was quantified by averaging the number of CD31-positive vessels in 10 randomly selected high-power microscopic fields (×400). The results are expressed as mean value ± standard error of the mean.

### RNA extraction and reverse transcription

Total RNA was extracted from the rat liver using a Maxwell® 16 LEV Simply RNA Tissue Kit (Promega K.K., Tokyo, Japan). Complementary DNA (cDNA) was synthesized from 1 µg of total RNA with Random Primers (Invitrogen, Thermo Fisher Scientific K.K., Yokohama, Japan) using an Omniscript® Reverse Transcription Kit (QIAGEN K.K., Tokyo, Japan).

### Quantitative PCR analysis

Quantitative polymerase chain reaction (PCR) was performed using a LightCycler® 2.0(DX400) Instrument (Roche Diagnostics, Tokyo, Japan) with a commercially available master mix (LightCycler® Taqman® Master, Roche Diagnostics, Tokyo, Japan). All primers were obtained from Sendai Wako Pure Chemicals, Ltd. (Sendai, Japan). The primer sequences were as follows: hepatocyte growth factor (HGF) (forward) 5′-CCATGTGGGACAAGAATATGGA-3′ and (reverse) 5′-TTCCGGCAGTAATTCTTAGTCA-3′; basic fibroblast growth factor (bFGF) (forward) 5′-TTGTGTCCATCAAGGGAGTG-3′ and (reverse) 5′-TTATTGGACTCCAGGCGTTC-3′; VEGF-A (forward) 5′-GCAGATCATGCGGATCAAAC-3′ and (reverse) 5′-GTTCTATCTTTCTTTGGTCTGCATT-3′; VEGF-B (forward) 5′-CTGGAGTGTGTGCCCATTG-3′ and (reverse) 5′-GCATTCACATTGGCTGTGTTC-3′; TGF-β1 (forward) 5′-CTGAACCAAGGAGACGGA-3′ and (reverse) 5′-CGTGGAGTACATTATCTTTGCTG-3′; collagen (Col)-3α1 (forward) 5′-CATGATGAGCTTTGTGCAATGT-3′ and (reverse) 5′-GGCTTCCAGACATCTCTAGACT-3′; β-actin (forward) 5′-CAAATGCTTCTAGGCGGACT-3′ and (reverse) 5′-GCGCAAGTTAGGTTTTGTCA-3′. Amplification was carried out in a final volume of 20 µL that included 5 µL of DNA template (0.05 µg/µL), 1 µL of each forward and reverse primer (final concentration: 0.5 µM), 1 µL hydrolysis probe (final concentration: 0.1 µM), and 4 µL of master mix. The cycling conditions were set as follows: 55 cycles at 95 °C for 10 seconds, 60 °C for 40 seconds, and 72 °C for 1 second. The mRNA expression of HGF, bFGF, VEGF-A, VEGF-B, TGF-β1, and Col-3α1 was normalized using the endogenously expressed housekeeping gene of β-actin as the internal control. The expression level of mRNA in each group was determined by comparing that of the control group.

### Biochemical analysis

Serum was prepared from blood after centrifugation at 3,000 r.p.m. for 15 minutes at 4 °C and filtered. After filtration, the values of liver and biliary enzymes, total protein, and albumin were determined in a biochemical automatic analyzer (DRI-CHEM 7000 V (Z), FUJIFILM Co., Tokyo, Japan). Results are expressed as mean value ± standard error of the mean.

### Statistics

Data are presented as means ± standard error of the mean. We adopted one-way analysis of variance with Tukey-Kramer’s honest significant difference multiple comparison test or the Steel-Dwass test to compare mean values, where appropriate. Statistically significant differences were defined as those with *p* < 0.05. Analyses of these data were performed using JMP Pro version 12.2.0 statistical software (SAS Institute Japan Ltd., Tokyo, Japan).

### Ethics

All animal procedures and protocols were approved by the institutional review board of the Center for Laboratory Animal Research of Tohoku University.

### Informed consent

This article does not contain any studies with human subjects.

### Animal study

All institutional and national guidelines for the care and use of laboratory animals were followed. The experimental protocol was approved by the Committee of Laboratory Animals according to the guidelines of Tohoku University, Sendai, Miyagi 980–8574, Japan.

## Results

### Histological analysis and quantification of liver fibrosis

**Figure 1:**
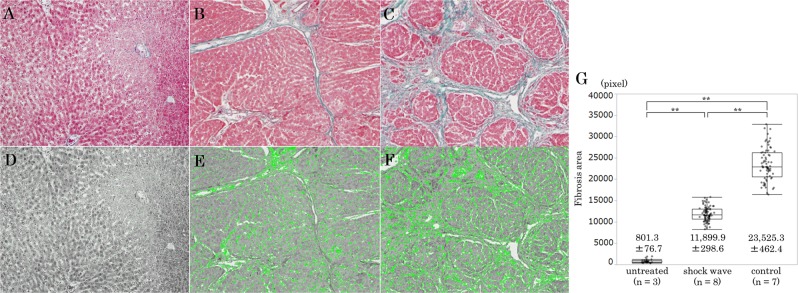
Photomicrographs of liver histology (**A–C**: Elastica-Masson staining, ×100; **D–F**: Binarized image, ×100; **G**: Quantification of liver fibrosis). The extent of fibrosis was quantified by binarizing to fibrous or not fibrous area using Win ROOF software (**D**: Untreated group; **E:** Shock wave group; **F**: Control group) in 10 microscopic fields (×100) per specimen in all rats. The quantified fibrous area was averaged for each group (**G**).

Staining of liver specimens with Elastica-Masson revealed that the livers of rats in the untreated group had almost no fibrosis and fibrosis was reduced in the shock wave group compared with the control group (Fig. 1A–C). Quantitative analysis of the fibrosis area using Win ROOF software in each group is shown in Fig. 1D–F; the fibrosis area is shown in green color. The area of fibrosis in the control group was significantly larger than that in the untreated group (23,525.3 ± 462.4 vs. 801.3 ± 76.7 pixels per field, *p* < 0.001) (Fig. 1G). The area of fibrosis in the shock wave group was significantly smaller than that in the control group (11,899.9 ± 298.6 vs. 23,525.3 ± 462.4 pixels per field, *p* < 0.001) (Fig. 1G).

### Immunohistochemistry

**Figure 2:**
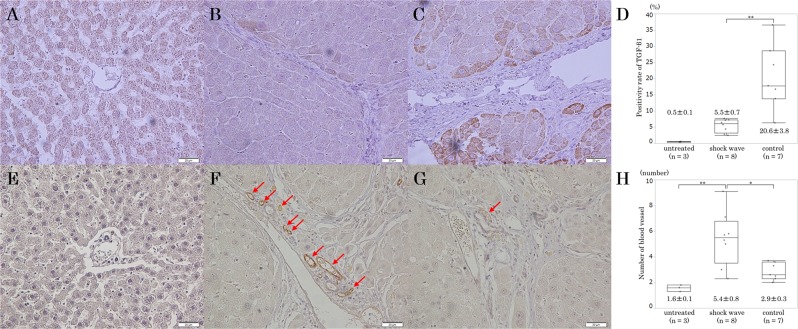
Immunohistochemical studies of transforming growth factor (TGF)-β1 (×400, **A**: Untreated group; **B**: Shock wave group; **C**: Control group; **D**: Quantification of the positivity expression rate of TGF-β1) and CD31 (×400, **E**: Untreated group; **F**: Shock wave group; **G**: Control group; **H**: Quantification of the number of CD31-positive vessels).

In specimens immunostained for TGF-β1, hepatic cells of rats in the control group were well stained compared with those of rats in the shock wave and untreated groups (Fig. 2A–C). The positivity expression rate of TGF-β1 in hepatic cells of untreated group was 0.5 ± 0.1% (Fig. 2D). In the shock wave group, the rate of TGF-β1 positivity was significantly lower than in the control group (5.5 ± 0.7% vs. 20.6 ± 3.8%, *p* = 0.018) (Fig. 2D).

Figure 2E–G shows immunohistochemical studies of CD31. No periportal CD31-positive vessels were seen in a high-power microscopic field (×400) of the untreated group (Fig. 2E). Several periportal CD31-positive vessels were seen in a high-power microscopic field of the shock wave group (Fig. 2F). In the control group, only one periportal CD31-positive vessel was seen in the same magnified view (Fig. 2G). The number of periportal CD31-positive vessels/field was significantly higher in the shock wave group than in the control group (5.4 ± 0.8 vs. 2.9 ± 0.3 pieces per field, *p* = 0.015) and the untreated group (5.4 ± 0.8 vs. 1.6 ± 0.1 pieces per field, *p* = 0.006) (Fig. 2H).

### Effects of shock wave on mRNA expression in the liver of factors associated with hepatic fibrosis

**Figure 3:**
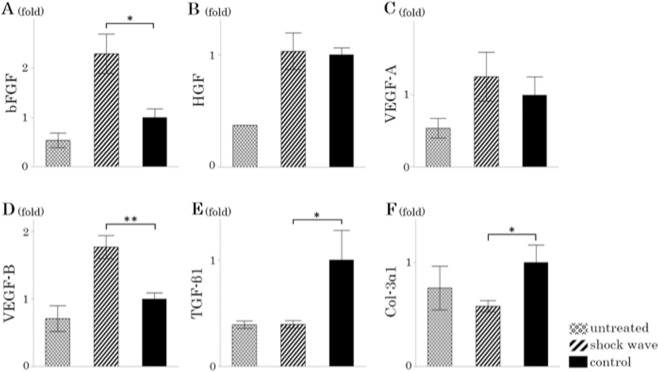
Expression level of mRNAs (**A**: Basic fibroblast growth factor (bFGF); **B**: Hepatocyte growth factor (HGF); **C**: Vascular endothelial growth factor (VEGF)-**A**; **D**: VEGF-**B**; **E**: Transforming growth factor (TGF)-β1; **F**: Collagen type III alpha 1 chain (Col-3α1)).

The mRNA expression level of bFGF was significantly higher in the shock wave group than in the control group (2.29-fold, *p* = 0.025) (Fig. 3A). The expression level of HGF mRNA was almost the same in the shock wave and control groups during this experiment (1.03-fold, *p* = 0.96) (Fig. 3B). Focusing on VEGF, the expression level of VEGF-A mRNA in the shock wave group tended to increase compared with that in the control group, but it should be noted that there was no difference between these groups (1.26-fold, *p* = 0.82) (Fig. 3C). The expression level of VEGF-B mRNA in the shock wave group was significantly higher than that in the control group (1.77-fold, *p* = 0.006) (Fig. 3D). On the other hand, the expression levels of TGF-β1 and collagen type III alpha 1 chain (Col-3α1) mRNAs in the shock wave group were significantly lower than those in the control group (0.40-fold, *p* = 0.018; 0.58-fold, *p* = 0.024) (Fig. 3E,F).

### Biochemical analysis

**Figure 4:**
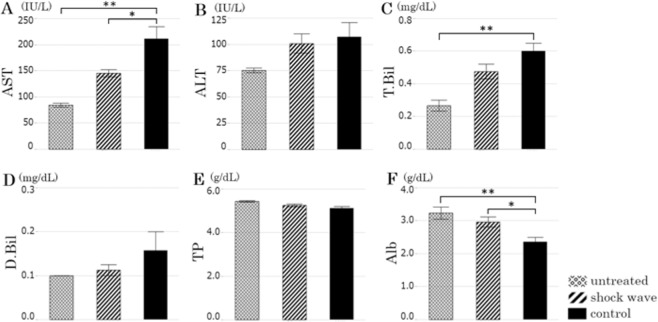
Data from biochemical examination (**A**: Aspartate aminotransferase (AST); **B**: Alanine aminotransferase (ALT); **C**: Total bilirubin (T-Bil); **D**: Direct bilirubin (D-Bil); **E**: Serum total protein (TP); **F**: Serum albumin (Alb)).

Aspartate aminotransferase (AST) levels were significantly higher in the control group than in the untreated group (211.0 ± 23.3 vs. 84.3 ± 3.8 IU/L, *p* = 0.001) and significantly lower in the shock wave group than in the control group (145.3 ± 7.0 vs. 211.0 ± 23.3 IU/L, *p* = 0.020) (Fig. 4A). Alanine aminotransferase (ALT) levels of untreated group were 75.3 ± 2.2 IU/L, and ALT levels were almost the same in the shock wave and control groups (100.9 ± 9.2 vs. 107.1 ± 13.7 IU/L, *p* = 0.91) (Fig. 4B). The AST/ALT ratio is significantly lower in the shock wave group than in the control group (1.49 ± 0.11 vs. 2.04 ± 0.14, *p* = 0.013). Although the differences were not significant, total bilirubin (0.48 ± 0.05 vs. 0.60 ± 0.05, *p* = 0.08) and direct bilirubin levels (0.11 ± 0.01 vs. 0.16 ± 0.04, *p* = 0.35) tended to be lower in the shock wave group than in the control group during this experiment (Fig. 4C,D). Serum total protein levels of untreated group were 5.4 ± 0.1 g/dL, shock wave group were 5.3 ± 0.1 g/dL and control group were 5.1 ± 0.1 g/dL; there were no differences among the three groups (Fig. 4E). Serum albumin was significantly lower in the control group than in the untreated group (2.4 ± 0.1 vs. 3.2 ± 0.2 g/dL, *p* = 0.015), but it was significantly higher in the shock wave group than in the control group (3.0 ± 0.2 vs. 2.4 ± 0.1 g/dL, *p* = 0.025) (Fig. 4F).

## Discussion

The irradiation conditions of the shock waves in this study were determined based on reports of LESW treatment^3,4,6,7^. Some previous studies reported that 0.1 mJ/mm^2^ (positive energy flux density) was effective for angiogenesis and inhibition of fibrosis in myocardial infarction model pigs and rats^3,6,7^. According to the manufacturer, the optimal focal point of the shock wave is within 10 mm width and 10 mm depth from the tip of the probe, and 0.1 mJ/mm^2^ (positive energy flux density) is equivalent to 0.25 mJ/mm^2^ (total energy flux density). In the present study, therefore, the output energy was set to 0.25 mJ/mm^2^. Since five spots from the epigastrium to the right hypochondrium were irradiated with the shock wave, the whole liver was irradiated considering liver volume of rat. Adverse events such as bleeding or death were not observed in the condition described above; thus, the LESW for the liver under these conditions was thought to be a safe and less invasive.

Liver fibrosis was significantly improved by LESW therapy in the present study. It has been reported that LESW suppressed TGF-β1 expression, which is known to promote left ventricular fibrosis and post-myocardial infarction remodeling^7^. TGF-β1 is overexpressed in all fibrotic tissues, and it induces collagen production^9^. The prominent role of TGF-β1 in fibrogenesis was observed, for example, when subcutaneous injection of purified TGF-β1 induced fibrotic lesions at the injection site^10^. It has been reported that neutralization of TGF with antiserum ameliorates experimental fibrosis in the liver^11^. TGF-β1 is involved in hepatic fibrosis and has fibrogenic action leading to transdifferentiation of hepatic stellate cells into myofibroblasts^12^. Activated myofibroblasts have been identified as collagen-producing cells^2^. Liver fibrosis is the excessive accumulation of extracellular matrix proteins, including type I, III, and IV collagen^2^. We investigated the expression level of Col-3α1 mRNA in this study, and it was significantly lower in the shock wave group than in the control group. These results suggest that LESW suppressed TGF-β1 expression, leading to suppression of collagen, such as Col-3α1, and improvement of hepatic fibrosis. Other types of collagen need to investigate in the future.

Angiogenesis is an essential process in liver regeneration, and VEGF is one of the main regulators of angiogenesis^13^. In the present study, the expression level of VEGF-B mRNA in the shock wave group was significantly higher than that in the control group. It is possible that VEGF-B is involved in angiogenesis after shock wave therapy for liver cirrhosis. However, the expression level of VEGF-A mRNA was not significantly different between the shock wave and control groups. It is possible that, in this study, the timing of the examination (day 4 after LESW therapy) was not adequate to detect VEGF-A expression, because the expression of VEGF-A mRNA peaks at 12 hours after shock wave treatment^14^. The expression level of VEGF-A in the shock wave group tended to increase compared with that in the control group, suggesting that it is also possible that VEGF-A is involved in the effect of shock wave therapy. Basic fibroblast growth factor is believed to induce angiogenesis by a direct effect on endothelial cells^15,16^. In the present study, the expression level of bFGF mRNA was significantly higher in the shock wave group than in the control group. This result may also suggest that the expression of bFGF was promoted by LESW. CD31 positive cells are markers of angiogenesis, which are expressed in endothelial cells of neovascular vessels^17^. In our study, the number of periportal CD31-positive vessels was significantly higher in the shock wave group than in the control and untreated groups. This result suggests that angiogenesis in liver was activated by LESW. These results suggest that LESW therapy increases the expression of angiogenic factor as VEGF and bFGF, and promote periportal vascularization. It has been reported that periportal angiogenesis is induced in hepatic regeneration^18^. It is possible that an increase in periportal vascularization caused by LESW results in regeneration of liver tissue.

It has been reported that AST rises with administration of carbon tetrachloride^19,20^. In this study, AST was significantly higher in the shock wave group than in the untreated group. It is considered that administration of carbon tetrachloride results in deterioration of liver function. On the other hand, LESW significantly decreased AST. Although the difference was not significant, other hepatobiliary functions tended to be lower in the shock wave group than in the control group. This result may suggest that LESW is helpful for improving liver function. It has been reported that the AST/ALT ratio is a dependable marker of liver cirrhosis, the ratio in the cirrhotic patients was higher than in the noncirrhotic patients^21^. As the AST/ALT ratio decreased by LESW therapy, it was thought that hepatic function was improved in this study.

Serum albumin was significantly lower in the control group than in the untreated group. In liver cirrhosis, albumin production is reduced, and hypoalbuminemia results in reduction of circulating blood volume, which may cause various complications^22,23^. The prognosis of cirrhotic patients with hypoalbuminemia is worse than that of patients with serum albumin of 3.5 g/dL or more^24^. It has been reported that administering serum albumin to cirrhotic patients with hypoalbuminemia corrects circulatory insufficiency and improves the survival rate^22,23^. Therefore, it is important to prevent decrease in serum albumin in cirrhotic patients. In this study, serum albumin was significantly higher in the shock wave group than in the control group. The result suggests that serum albumin was improved by LESW therapy, and that LESW therapy may be useful for improving the general condition of cirrhotic patients.

We determined the irradiation conditions of shock wave treatment, such as output level and the number of impulses, based on several previous reports^3,4,6,7^. However, these previous reports described the effects of shock wave treatment on ischemic heart disease. There might be more optimal irradiation condition of shock wave treatment for liver cirrhosis. We also determined the collection date based on a previous report that the expression of growth factors such VEGF or HGF peaks at 72 to 120 hours of liver regeneration after hepatectomy^25^. It was considered to change the timing of sample collection, because liver regeneration caused by shock wave might have a different course than liver regeneration after hepatectomy. In the present study, LESW therapy was performed only once for liver cirrhosis. There might be more effects when multiple treatments are performed. Further studies are needed to clarify these issues.

## Conclusion

This study investigated the effect of LESW on the liver in a rat model of drug-induced liver cirrhosis. The novel finding of the study was that LESW therapy improved hepatic fibrosis, hepatic function, and hepatic angiogenesis, accompanied by down-regulation of TGF-β1 and up-regulation of angiogenic factors such as bFGF and VEGF-B. This animal study suggests that LESW has the potential to be a safe and noninvasive approach for treatment of liver cirrhosis in the future.
